# Editorial: Microtubule-associated molecular motors: Transport mechanisms and role in disease

**DOI:** 10.3389/fcell.2022.1106435

**Published:** 2022-12-07

**Authors:** Virupakshi Soppina, Xin Xiang, Senthil Arumugam

**Affiliations:** ^1^ Discipline of Biological Engineering, Indian Institute of Technology Gandhinagar, Gandhinagar, Gujarat, India; ^2^ Department of Biochemistry and Molecular Biology, The Uniformed Services University of the Health Sciences-F. Edward Hébert School of Medicine, Bethesda, MD, United States; ^3^ Faculty of Medicine, Nursing, and Health Sciences, Monash Biomedicine Discovery Institute, Monash University, Melbourne, VIC, Australia

**Keywords:** microtubules, dynein, kinesin, transport, motors

Long-distance transport is a fundamental process of life from single-cell amoeba to multicellular animals. Cells have a highly organized and efficient microtubule-based transport system called “intracellular transport” which dependent on microtubule-associated molecular motor proteins. Microtubules are self-assembled filaments that extend from the cell center (known as “minus-end”) towards the cell periphery (known as “plus-end”) and serve as tracks for molecular motors during intracellular transport. Members of kinesin and dynein families are mechanochemical enzymes that hydrolyze ATP to generate force and take steps along the microtubules while transporting cellular cargo. Most kinesins transport cargo towards the plus-end of microtubule (called as anterograde transport), whereas dyneins walk towards the minus-end of microtubule (called as retrograde transport).

Molecular motor proteins of the kinesin and dynein families have been established to play critical roles in myriads of cellular processes such as intracellular and axonal transport, viral trafficking, endocytosis, development, signaling, and homeostasis. Cytoplasmic dynein is a highly versatile motor that transports various cargoes toward the minus ends of microtubules (Reck-Peterson et al., 2018; Olenick and Holzbaur, 2019). It also plays roles in mitosis, including mitotic spindle-pole focusing (Borgal and Wakefield, 2018; Kiyomitsu and Boerner, 2021). Kinesins belong to a superfamily containing about 14 members. Unlike kinesin-1 and kinesin-3, which drive the movements of membranous cargos, mitotic kinesins such as kinesin-5 and kinesin-14 are involved in organizing mitotic spindles (Vale and Fletterick, 1997; Verhey et al., 2011). The initial discovery of dynein and kinesin (Gibbons and Rowe, 1965; Vale et al., 1985; Paschal et al., 1987; Vallee et al., 1988) has inspired intense research to understand the mechanisms and cellular functions of these motors. Yet the molecular mechanisms and regulation of these motor proteins are still not completely understood, especially in direct physiological relevance. Several studies have demonstrated that the presence and simultaneous activity of the same- or opposite-polarity motor proteins are critical for generating large collective force and motility, which is essential for transporting cellular cargo (Soppina et al., 2009). This process is tightly regulated by cellular physiology, signaling functions and proteins, including Rab GTPases, adaptor proteins and microtubule post-translational modifications. More recently, regulatory factors and regulatory mechanisms of the motor proteins have also become fascinating topics in the motor field. Abnormalities in cytoskeletal elements, defects or alterations in their functions/regulation are often associated with diseases that include neurodegenerative and developmental diseases, cancer and ciliopathies (Hirokawa et al., 2010; Soppina et al., 2014; Soppina et al., 2022a; Soppina et al., 2022b). Yet, the molecular basis of these pathologies has not been fully understood.

The aim of this Research Topic was to cover recent progress, novel research findings and limitations in the microtubule-based intracellular cargo trafficking field to understand the mechanism and regulations of cargo trafficking and their implications in human pathologies. In most eukaryotes, cytoplasmic dynein, a multisubunit protein complex, serves as the primary minus end-directed microtubule motor for intracellular transport. Interestingly, land plants lack dynein, but they have a large number of kinesin-14s, which is critical for spindle organization in acentrosomal plant cells (Yamada and Goshima, 2017; Liu and Lee, 2022). In this context, Hotta et al. present original research results suggesting that ATK1 and ATK5, two kinesin-14s in Arabidopsis thaliana, establish convergent spindle poles by forming oligomeric complexes that can translocate microtubules toward the spindle poles.

Motor-mediated cargo transport in different cell types still needs to be better understood. In this context, Hazim and Williams review the functions of cytoplasmic dynein and kinesins in retinal pigment epithelium (RPE), polarized epithelial cells with a special microtubule-organization pattern, and how retinal pathology may be linked to defects in motor functions.

Lipid droplets (LDs) are cellular organelles involved in diverse cellular processes, including energy storage, cellular signaling, development and building cell membranes. Studies are beginning to uncover how LDs interact with other cellular compartments and how these interactions play a role in lipid metabolism. Singh et al. review the biology of lipid droplets in liver cells, how motor proteins such as dynein and kinesin-1s are recruited onto these droplets to affect the interactions of lipid droplets with particular organelles, and how they affect liver cell physiology. Molecular definitions of organelle-organelle interactions, especially in disease-relevant systems will reveal new mechanisms and the review by (Singh et al.) effeceintly summarises this approach.

Recycling endosomes are formed from membrane tubulations emanating from early endosomes that undergo scission. A lot remains to be understood about their role in cargo sorting, molecular players and processes like endosomal escape, that take place at membrane tubulations. Thankachan and Gangi Setty review the function of Kif13A, a member of the kinesin-3 family, in biogenesis and transportation of recycling endosomes, how its function affects membrane dynamics and how it is regulated.

How various motor proteins are regulated is an area of intense investigation. In the cytoplasmic dynein field, new knowledge on several key regulatory factors such as dynactin, LIS1 (lissencephaly-1) and cargo adapters have been covered in multiple reviews (Schroer, 2004; Reck-Peterson et al., 2018; Olenick and Holzbaur, 2019; Canty and Yildiz, 2020; Markus et al., 2020; Xiang and Qiu, 2020; Cason and Holzbaur, 2022). In this context, Garrott et al. review our current knowledge on two less well-studied dynein regulators, Nde1 and Ndel1, paralogs of LIS1-binding proteins, which highlight the need for future work to further understand these more mysterious regulatory factors.

In general, non-cargo-bound kinesins are autoinhibited through an intramolecular interaction between the tail-motor domain or motor-stalk interaction and prevent futile ATP consumption and microtubule crowding (Verhey and Hammond, 2009; Siddiqui and Straube, 2017). Cargo binding releases motor inhibition and enables cellular cargo transport. However, cargo transport is dynamic and regulated by multiple factors (Guedes-Dias and Holzbaur, 2019; Koppers and Farías, 2021). Among other mechanisms, phosphorylation of motors, motor adaptors, and cargo can achieve such regulation. In this context, Kumari and Ray review the knowledge of how phosphorylation is involved in regulating different types of kinesins, including kinesin-1, kinesin-2 and kinesin-3. Together, these papers in this topic highlight the need to understand further how motor proteins function in different cell types or biological contexts, how motor-cargo interaction is achieved for vesicles/organelles, and how motor activities are regulated.
